# Combined GWAS and ‘guilt by association’-based prioritization analysis identifies functional candidate genes for body size in sheep

**DOI:** 10.1186/s12711-017-0316-3

**Published:** 2017-04-28

**Authors:** Antonios Kominakis, Ariadne L. Hager-Theodorides, Evangelos Zoidis, Aggeliki Saridaki, George Antonakos, George Tsiamis

**Affiliations:** 1 Department of Animal Science and Aquaculture, Agricultural University of Athens, Iera Odos 75, 11855 Athens, Greece; 2 Department of Environmental and Natural Resources Management, University of Patras, Seferi 2, 30100 Agrinio, Greece; 3 Agricultural and Livestock Union of Western Greece, 13rd Km N.R. Agrinio-Ioannina, 30100 Lepenou, Greece

## Abstract

**Background:**

Body size in sheep is an important indicator of productivity, growth and health as well as of environmental adaptation. It is a composite quantitative trait that has been studied with high-throughput genomic methods, i.e. genome-wide association studies (GWAS) in various mammalian species. Several genomic markers have been associated with body size traits and genes have been identified as causative candidates in humans, dog and cattle. A limited number of related GWAS have been performed in various sheep breeds and have identified genomic regions and candidate genes that partly account for body size variability. Here, we conducted a GWAS in Frizarta dairy sheep with phenotypic data from 10 body size measurements and genotypic data (from Illumina ovineSNP50 BeadChip) for 459 ewes.

**Results:**

The 10 body size measurements were subjected to principal component analysis and three independent principal components (PC) were constructed, interpretable as width, height and length dimensions, respectively. The GWAS performed for each PC identified 11 significant SNPs, at the chromosome level, one on each of the chromosomes 3, 8, 9, 10, 11, 12, 19, 20, 23 and two on chromosome 25. Nine out of the 11 SNPs were located on previously identified quantitative trait loci for sheep meat, production or reproduction. One hundred and ninety-seven positional candidate genes within a 1-Mb distance from each significant SNP were found. A guilt-by-association-based (GBA) prioritization analysis (PA) was performed to identify the most plausible functional candidate genes. GBA-based PA identified 39 genes that were significantly associated with gene networks relevant to body size traits. Prioritized genes were identified in the vicinity of all significant SNPs except for those on chromosomes 10 and 12. The top five ranking genes were *TP53*, *BMPR1A*, *PIK3R5*, *RPL26* and *PRKDC*.

**Conclusions:**

The results of this GWAS provide evidence for 39 causative candidate genes across nine chromosomal regions for body size traits, some of which are novel and some are previously identified candidates from other studies (e.g. *TP53*, *NTN1* and *ZNF521*). GBA-based PA has proved to be a useful tool to identify genes with increased biological relevance but it is subjected to certain limitations.

**Electronic supplementary material:**

The online version of this article (doi:10.1186/s12711-017-0316-3) contains supplementary material, which is available to authorized users.

## Background

Body size (BS) is a typical quantitative (or complex) trait that shows continuous variation. According to the infinitesimal model of Fisher [1], traits such as BS are genetically controlled by an infinite number of loci, each with an infinitesimal effect. More recently, the infinitesimal model has gradually been replaced by hundreds or thousands of discrete genes each with many mutable sites and (possible) segregating mutations [2].

The genetic basis of BS has been investigated in cattle (e.g. [3]) and dogs (e.g. [4]). In non-giant dog breeds, Rimbault et al. [5] showed that about half of the variance in BS can be explained by seven single nucleotide polymorphisms (SNPs) that lie in close proximity to the *GHR*, *HMGA2*, *SMAD2*, *STC2*, *IGF1* and *IGF1R* genes. In humans, until recently, more than 20 genome-wide association studies (GWAS) identified over 400 candidate genes associated with human stature [6]. Interestingly, in human populations, mutations with an intermediate effect cannot be or are poorly detected by current approaches, whereas such mutations can occasionally be detected in domestic animals when artificial selection or genetic drift increases their frequency [2].

In sheep, BS has been extensively recorded for years because it is an important indicator of growth and health, it affects feeding and housing management and has consequences on this species’ environmental adaptation [2]. However, the causative loci that contribute to the genetic variation of this trait, remain largely unknown. This may be attributed to the incomplete information on the sheep genome with only about 700 genes known before the release of Ovis_aries_v3.1 reference genome sequence in 2012 [7]. Since then, there has been considerable progress and the latest assembly of the sheep genome (Oar_v4.0, [8]), which is based on the dataset from a Texel ewe with a 166-fold coverage, has a total assembled length of 2.61 Gb, and the current annotation [9] includes 20,645 protein-coding genes. Another obstacle in the elucidation of the molecular basis of BS in sheep is the insufficient number of animals with both phenotypes and high-density genotyping data.

High-throughput SNP genotyping has been used to detect signatures of selection or perform GWAS that aim at identifying loci and genes involved in the variation of BS in sheep. Randhawa et al. [10] identified a signature of selection that included the *LRP4* gene on *Ovies aries* chromosome OAR25 and is associated with bone growth. Using the same methodology, Kijas et al. [11] identified three genomic regions that spanned the genes *NPR2* (OAR1), *HMGA2* (OAR5) and *BMP2* (OAR18) and were associated with skeletal morphology and BS. A GWAS reported by Zhang et al. [7] detected 36 SNPs that were significantly associated at the chromosome-wise level with seven growth and meat production traits and 10 of these SNPs reached genome-wise significance. These authors identified candidate genes based on the chromosomal position of these SNPs, with genes that either harbored a significant SNP i.e. *MEF2B*, *RFXANK*, *CAMKMT*, *TRHDE* and *RIPK2*, or were located in close proximity of a significant SNP, i.e. *GRIM1*, *POL*, *MBD5*, *UBR2*, *RPL7* and *SMC2*. Al-Mamun et al. [12] identified a region on OAR6 that harbored three candidate genes *LAP3*, *NCAPG* and *LCORL* with the latter being associated with height in humans and cattle. Most recently, by combining 56 single GWAS for carcass composition in a meta-analysis, Bolormaa et al. [13] detected a group of 23 SNPs with pleiotropic effects on mature size, which are associated with size and fatness traits in humans and cattle. In addition to DNA sequence polymorphisms, epigenetic regulation of gene expression, mainly via DNA methylation, may also contribute to the phenotypic variation in BS in sheep. Cao et al. [6] detected significant correlations between the genetic variability at CpG sites of methylation and RNA expression of the *BMPR1B*, *SMAD1*, *TSC1* and *AKT1* genes, which are associated with BS variability in Mongolian sheep breeds.

Many of the published GWAS in sheep and other species provide potentially interesting findings in the form of large lists of candidate genes, even if the statistical power is limited in some cases. Such large lists do not facilitate downstream validation due to their size and the lack of prioritization of the positional candidate genes based on their likelihood of harboring true causal mutations that contribute to the trait’s variability. The large number of candidate genes derived from GWAS for quantitative traits requires computational approaches that can assess the functional relevance of the positional candidate genes and prioritize these accordingly, especially when the genes are insufficiently annotated, as is often the case. One of the widely used principles for elucidating the function of un-annotated genes, i.e. for gene function prediction, is the guilt-by-association (GBA) principle [14]. The GBA principle states that genes that are involved in the same biological processes tend to be associated (or possess similar properties e.g. similar expression patterns), which allows to statistically infer previously unknown functions of a gene based on some prior knowledge about other genes and association data [15]. Gene networks based on the GBA principle have been successfully implemented in disease-gene discovery (e.g. [16]) and gene function prediction in various species [17, 18].

BS can be described by phenotypic measurements and visual assessments. One (e.g. withers height) or more measurements (e.g. width at different anatomical structures) that are usually inter-correlated are used. In such cases, the standard way to perform a GWAS is to apply a multivariate approach. However, when numerous traits are examined, the application of a multivariate approach becomes infeasible. A second option is to perform GWAS on the individual traits and then integrate GWAS information using network inference algorithms (e.g. association weight matrix, [19]) in an attempt to identify key regulatory elements and generate gene networks of complex traits. A third approach is to apply a trait reduction method such as principal component analysis (PCA), which determines a few, meaningful uncorrelated components of the traits i.e. the principal components (PC) that explain a significant part of the variance of the original traits [20]. After constructing the PC, GWAS are carried out on the individual PC, which results in the detection of significantly associated markers. The PC approach has two major advantages: (1) no multivariate analysis is needed since PC are uncorrelated, and (2) the power of GWAS can be enhanced since PC are composed of multiple traits.

In this study, first we attempted to describe the phenotypic variation of 10 objectively measured BS traits in the Frizarta dairy sheep breed by a minimum number of independent variables (PC). The PC were then associated with genotypic data obtained with the Illumina OvineSNP50 BeadChip to identify significant quantitative trait nucleotides (QTN) and candidate genes for each PC. Finally, a GBA-based gene prioritization method was applied to identify the most plausible functional candidate genes for BS traits.

## Methods

### Experimental animals

In this study, we used data on Frizarta officially registered ewes. This sheep breed is located in the north-western part of Greece and more specifically in the Arta and Agrinio districts. It is a synthetic breed that was formed by the introduction of East Friesian rams, which were extensively mated to ewes of the local breed during the years from 1961 to 1967 and from 1968 to 1982. The Frizarta breed is well adapted to the local climate of the area with mild winters, high rainfall and high relative humidity. Currently, about 11,000 ewes, dispersed in 87 herds, are officially registered (personal communication, Center of Animal Genetic Improvement of Athens, June 2015). Milk yield using the official A4 method [21] and litter size were recorded under the responsibility of two Cooperatives, located at Agrinio and Arta, respectively. Specifically, the Cooperative of the Agricultural and Livestock Union of Western Greece (ALUWG) located at Agrinio, is responsible for the official recording of 3746 ewes dispersed in 28 herds. Since 2010, trait recording has been extended to milk quality traits such as fat content, protein content, lactose and somatic cell count as well as udder and teat morphology traits. Recently, the Cooperative has undertaken initiatives towards the application of modern selection schemes, including genomic selection.

### SNP genotyping and quality control

A total of 524 dairy ewes of the Frizarta breed, distributed in seven of the 28 herds of the ALUWG were randomly selected for genotyping. Ewes are kept under an intense production system, with standardized conditions and feeding regime. DNA was extracted from blood samples of 524 dairy ewes using the NucleoSpin Blood kit (Macheray-Nagel). Genotyping using the Illumina OvineSNP50 BeadChip was performed commercially at Neogen Europe, Ltd. Among the 524 original samples, one sample could not be genotyped. Quality control (QC) of the remaining 523 genotypes was assessed in two stages, first on an ‘individual’ and second on a ‘marker’ basis. On the first level, samples were removed if they had: (1) a call rate lower than 0.95 and (2) an overall autosomal heterozygosity rate that fell outside the 1.3 inter-quartile range (0.346–0.389). Based on these criteria, 503 animals (samples) remained. Marker QC removed markers (1) with a call rate lower than 0.95, (2) with a minor allele frequency (MAF) lower than 0.05, (3) that deviated from Fisher’s Hardy–Weinberg equilibrium (HWE) p < 0.0001 and (4) that showed linkage disequilibrium (LD) r^2^ values greater than 0.50 within windows with a 50-kb inter-marker distance. The above criteria followed the guidelines of Anderson et al. [22]. Specifically, SNP pruning was applied to reduce correlations between SNPs due to LD [23]. Finally, only mapped SNPs and SNPs located on autosomes were considered. Thus, from the original 54,013 SNPs, 43,110 remained for the GWAS.

### Body measurements

Four hundred and eighty of the genotyped animals were visited during May 2014 to record measurements on 10 BS traits, i.e. withers height (WH), back height (BH), hip bone (hook) height (HH), body length (BL), chest girth (CG), shoulder width (SW), thorax width (TW), hip bone (hook) width (HW), rump width (RW) and pin bone width (PW), using measuring tape, compass and staff. After data inspection, the final dataset included 459 ewes with full records on all BS traits. Descriptive statistics for these 10 body size traits are in Table 1.

**Table 1:** Descriptive statistics for 10 body size traits on Frizarta dairy ewes

Trait	Abbreviation	Mean ± SEM	SD
Wither height (cm)	WH	71.74 ± 0.13	2.94
Back height (cm)	BH	72.28 ± 0.15	3.33
Hip bone (hook) height (cm)	HH	73.22 ± 0.15	3.36
Body length (cm)	BL	80.03 ± 0.24	5.20
Chest girth (cm)	CG	109.46 ± 0.34	7.48
Shoulder width (cm)	CW	19.90 ± 0.11	2.39
Thorax width (cm)	TW	24.28 ± 0.11	2.50
Hip bone (hook) width (cm)	HW	21.39 ± 0.08	1.79
Rump width (cm)	RW	19.19 ± 0.07	1.54
Pin bone width (cm)	PW	13.42 ± 0.06	1.25

### Principal components analysis

All BS traits followed a normal distribution and analysis of variance (ANOVA) showed that herd (seven classes), lactation number (six classes: 1, 2, 3, 4, 5 and ≥6) and lambing month (six classes: 1, 2, 9, 10, 11 and 12) were statistically significant effects. Traits were then adjusted for these effects based on the least square estimates of each class effect and for each trait. A principal component analysis (PCA), conducted with SAS (9.2) on the adjusted traits was then applied to determine the new uncorrelated variables, i.e. the principal components method was used to extract the components followed by a varimax rotation to obtain orthogonal (uncorrelated) components. Selection of the retained components was based on the following criteria: eigenvalues greater than 1, the Cattell’s scree test and finally interpretable factors. In the interpretation of the rotated factor pattern, a variable was said to load on a given component if the factor loading was equal to 0.40 or more for that component, and was less than 0.40 for the other. The Kaiser-Meyer Olkin (KMO) test of overall sampling adequacy was used to test for appropriateness of conducting PCA with this sample [24]. The KMO test provided a value of 0.82 for the set of variables, which is well above the value of 0.60 that is considered tolerable to explain the correlations between the variables [24].

### Marker association analysis

A multi-locus mixed (additive) model (MLMM) using the MLMM algorithm in [25] with a forward and backward stepwise approach to select SNPs as fixed effect covariates was used. A kinship matrix between samples was also calculated based on the identity-by-state (IBS) distance of the SNPs and included as a random effect in the mixed model. This analysis was carried out with the SNP and Variation Suite v8.3.4 (Golden Helix, Inc. 2015).

PC data were analyzed using the following mixed model:

$${\mathbf{y}} = {\mathbf{X \beta}} + {\mathbf{Zu}} + {\mathbf{e}},$$

where **y** is the vector of PC1, PC2 or PC3, **β** is the vector of the fixed effect for the minor allele of the SNP to be tested for association, **u** is the vector of random polygenic effects and **e** is the vector of random residuals. **X** is the incidence matrix relating observations to SNP effects with elements coded as 0, 1 or 2 for homozygous reference alleles, heterozygous alleles, and homozygous alternate alleles, respectively, and **Z** is the incidence matrix relating observations to the random polygenic random effects.

The random effects were assumed to be normally distributed with zero means and the following covariance structure:

$$Var\left[ {\begin{array}{*{20}c} {\mathbf{u}} \\ {\mathbf{e}} \\ \end{array} } \right] = \left[ {\begin{array}{*{20}l} {{\mathbf{G}}\upsigma_{{\mathbf{u}}}^{2} } \hfill & 0 \hfill \\ 0 \hfill & {{\mathbf{I}}\upsigma_{{\mathbf{e}}}^{2} } \hfill \\ \end{array} } \right],$$

where $\upsigma_{{\mathbf{u}}}^{2}$ and $\upsigma_{{\mathbf{e}}}^{2}$ are the polygenic and error variance components, **I** is the nxn identity matrix, and **G** is the n × n genomic relationship matrix [26] with elements of the pairwise relationship coefficient using all 43,110 SNPs. The genomic relationship coefficient between two individuals *j* and *k*, was estimated as follows:

$$\frac{1}{{{\text{n}}_{\upphi} }}\mathop \sum \limits_{i = 1}^{{{\text{n}}_{\upphi} }} \frac{{\left( {{\text{x}}_{ij} - 2p_{i} } \right)\left( {{\text{x}}_{ik} - 2p_{i} } \right)}}{{2p_{i} \left( {1 - 2p_{i} } \right)}},$$

where ${\text{n}}_{\upphi}$ is the number of SNPs (43,110), ${\text{x}}_{ij}$ and ${\text{x}}_{ik}$ the numbers (0, 1 or 2) of the reference allele(s) for the *i*th SNP of the *j*th and *k*th individuals, respectively, and *p*_*i*_ is the frequency of the reference allele [26].

### Quantile–quantile plots and estimation of the genomic inflation factor

Q–Q plots were used to analyze the extent to which the observed distribution of the test statistic followed the expected (null) distribution. This analysis along with the estimation of the genomic inflation factor ($\uplambda_{\text{gc}}$) was done to assess potential systematic bias due to population structure or to the analytical approach [27]. $\uplambda_{\text{gc}}$ was estimated as the median of the Chi squared test statistics of the nominal p values, divided by the expected median of the Chi squared distribution. The median of a Chi squared distribution with one degree of freedom is 0.4549. If the data follow the standard Chi squared distribution, the expected $\uplambda_{\text{gc}}$ value would be 1. If the $\uplambda_{\text{gc}}$ value is greater than 1, it provides evidence for some systematic bias.

### Multiple-testing correction

p values of SNPs obtained from the mixed model analysis were first corrected for multiple comparison by applying the Bonferroni correction method, which assumes independency between SNPs. To make this correction method more acceptable, some SNP pruning was applied but not all of the remaining SNPs tested for association remained independent i.e. uncorrelated, due to LD, which made the Bonferroni correction a rather conservative approach. To overcome this problem, the false-discovery rate (FDR) procedure [22] as an alternative correction method for multiple comparisons was also used with a FDR *p* value less than 0.10 considered as being significant. Using this method, a threshold p value of 0.10 would mean that on average 10% of the observed results would be false positives. Both correction methods were applied by using the MULTTEST procedure in SAS (2015).

### Proportion of variance explained

The proportion of variance explained by SNP *k* (${\text{pve}}_{k}$) was calculated as:

$${\text{pve}}_{k} = \frac{{{\text{mrss}}_{{{\text{h}}0}} - {\text{mrss}}_{k} }}{{{\text{mrss}}_{{{\text{h}}0}} }},$$

where ${\text{mrss}}_{{{\text{h}}0}}$ is the Mahalonobis root sum of squares (${\text{mrss}}$) of the null hypothesis and ${\text{mrss}}_{k}$ is the same for marker *k*.

### Related QTL

To investigate if the significant SNPs detected in this study were within the range of previously identified QTL for relevant traits, we searched for meat or production QTL in the SheepQTLdb [28] within a 1-Mb region on both sides of each significant SNP.

### Identification of positional candidate genes

Since in this breed levels of LD were higher than 0 between markers at distances up to 1 Mb (results not shown), we searched in 1-Mb regions around a significant SNP for candidate genes, which could be involved in the observed significant associations with the PC. We also used this distance range to alleviate any negative effect of the pruning of SNPs during their selection on the identification of causal variants. The exact positions of the annotated genes were extracted from the latest sheep genome Oar_v4.0 assembly [29] along with the NCBI annotation release 102 of the sheep genome [30].

### Functional characterization of positional candidate genes and gene prioritization

We first searched for human annotated genes that are associated with the ‘stature’ phenotype using the *GUILDify* web application [31]. We used the ‘stature’ description since it is the most common term used to describe height in humans. The *GUILDify* application exploits the physical interactions that occur between the proteins encoded by the genes and the GBA principle (proximity in the network to known components of a process) in the protein–protein interaction network (PPIN) to uncover phenotype-gene associations. The initial phenotype-gene associations are retrieved via free text search in biological databases. *GUILDify* uses network-topology based prioritization algorithms in GUILD to score the relevance of gene products with respect to given keywords. First, the BIANA knowledge base, which integrates data from publicly available major data repositories, was queried for gene products associated with the keyword ‘stature’. Next, the gene products retrieved were fed to a species-specific interaction network (created using BIANA) as seed proteins. Finally, a score of relevance for each gene product in the network was calculated by the prioritization algorithm based on network topology. Following this approach, 200 of the most highly ranked genes were selected based on the GUILD score (>0.147) for the keyword ‘stature’ in humans.

Next, gene prioritization analysis (PA) of the positional candidate genes was done, based on their functional similarity to a training gene list that included genes identified from the previous step (gene functional characterization). This analysis was carried out with the ToppGene portal [32]. This portal performs functional annotation-based candidate gene prioritization using fuzzy-based similarity measures to compute the similarity between any two genes based on semantic annotations. In our study, we used the following semantic annotations: Human and Mouse Phenotype plus GO plus Pathway. A p value for each annotation of a test gene was derived by random sampling of 5000 genes from the whole genome and these partial p values were combined into an overall score using statistical meta-analysis. Gene ranking was performed by applying the PPIN-based candidate gene prioritization and the K-Step Markov method. For gene prioritization, there were 190 training genes and 160 test genes (positional candidate genes). Not all of the 197 positional candidate genes were included in the analysis because some of these, mainly LOC genes, could not be mapped to human homologs. Genes with an overall p value lower than 0.05 were considered as significant.

## Results

### Principal component analysis

Results of the PCA are in Table 2. Following the eigenvalue criterion, only the first three components were retained for orthogonal rotation. The first (PC1), second (PC2) and the third components (PC3) explained 0.452, 0.182, and 0.106 of the total variance for the 10 traits, respectively. Combined together PC1, PC2 and PC3 accounted for 74% of the total variance (Table 2). Six measurements (CG, SW, TW, HW, RW and PW) were found to load on PC1 with factor loadings ranging from 0.61 (SW) to 0.86 (HW). This component was interpreted as the ‘width dimension’ factor. Another three measurements i.e. WH, BH and HH were found to load on PC2 with the highest factor loadings (>0.90). This component could be interpreted as the ‘height dimension’ factor. Finally, PC3 was formed by BL and was thus labeled as the ‘length dimension’ factor.

**Table 2:** Rotated factor pattern (×10) from principal component analysis on the 10 body traits

Trait	Component factor loading		
	1	2	3
Wither height	20	92^a^	9
Back height	16	95^a^	4
Hip bone (hook) height	19	94^a^	−1
Body length	20	10	81^a^
Chest girth	66^a^	32	23
Shoulder width	61^a^	11	35
Thorax width	81^a^	6	29
Hip bone (hook) width	86^a^	15	5
Rump widt	73^a^	18	−11
Pin bone width	63^a^	32	−40
Eigenvalue	4.521	1.824	1.058
Proportion of variance explained (of total)	0.452	0.182	0.106

^a^Traits with factor loadings >0.40

### Quantile–Quantile plots and estimation of $\uplambda_{\text{gc}}$

**Fig. 1:**
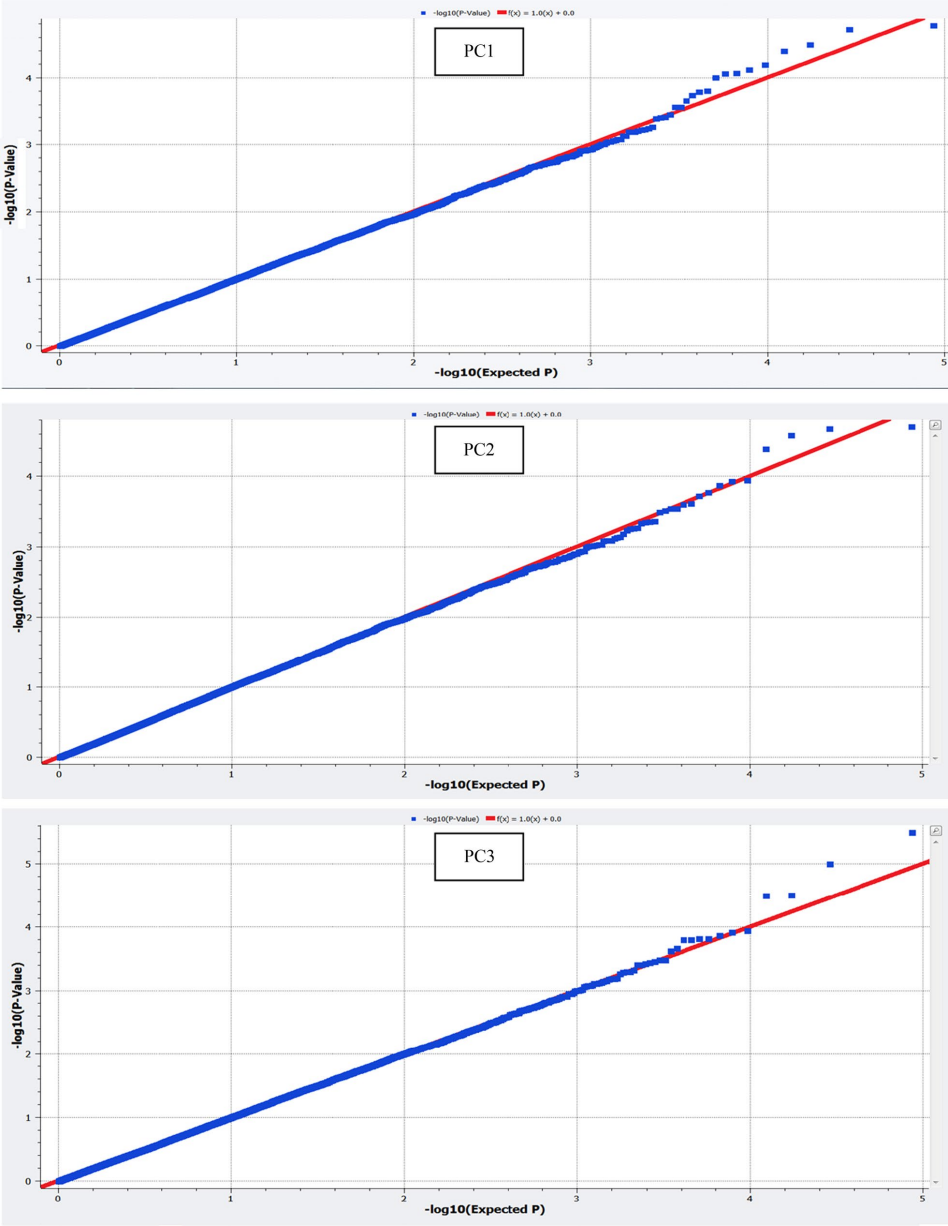
Quantile-Quantile plots for principal components (PC) 1, 2 and 3. *Blue dots* represent the −log10(p value) of the entire study and the *red lines* represent the expected values for the null hypothesis of no association

Figure 1 shows the QQ plots of the expected and the observed p values (−log10 p values) of all SNPs across the three PC. The genomic inflation factors ($\uplambda_{\text{gc}}$) for the three PC were equal to 1.057, 1.051 and 1.059, respectively. According to Kang et al. [33], $\uplambda_{\text{gc}}$ values that lie outside of the conservative 95% confidence interval (0.992 to 1.008) denote dependency of SNPs. However, as the QQ plots clearly show, there is no evidence of any systematic bias due to population structure or analytical approach in our case. As Yang et al. [21] emphasize in their paper, it is reasonable to expect large genomic inflation factors for purely polygenic traits such as those examined here in the absence of any systematic bias. The QQ plots also show that some SNPs depart from the expected probability and thus might be associated with the respective PC.

### Significant SNPs

**Fig. 2:**
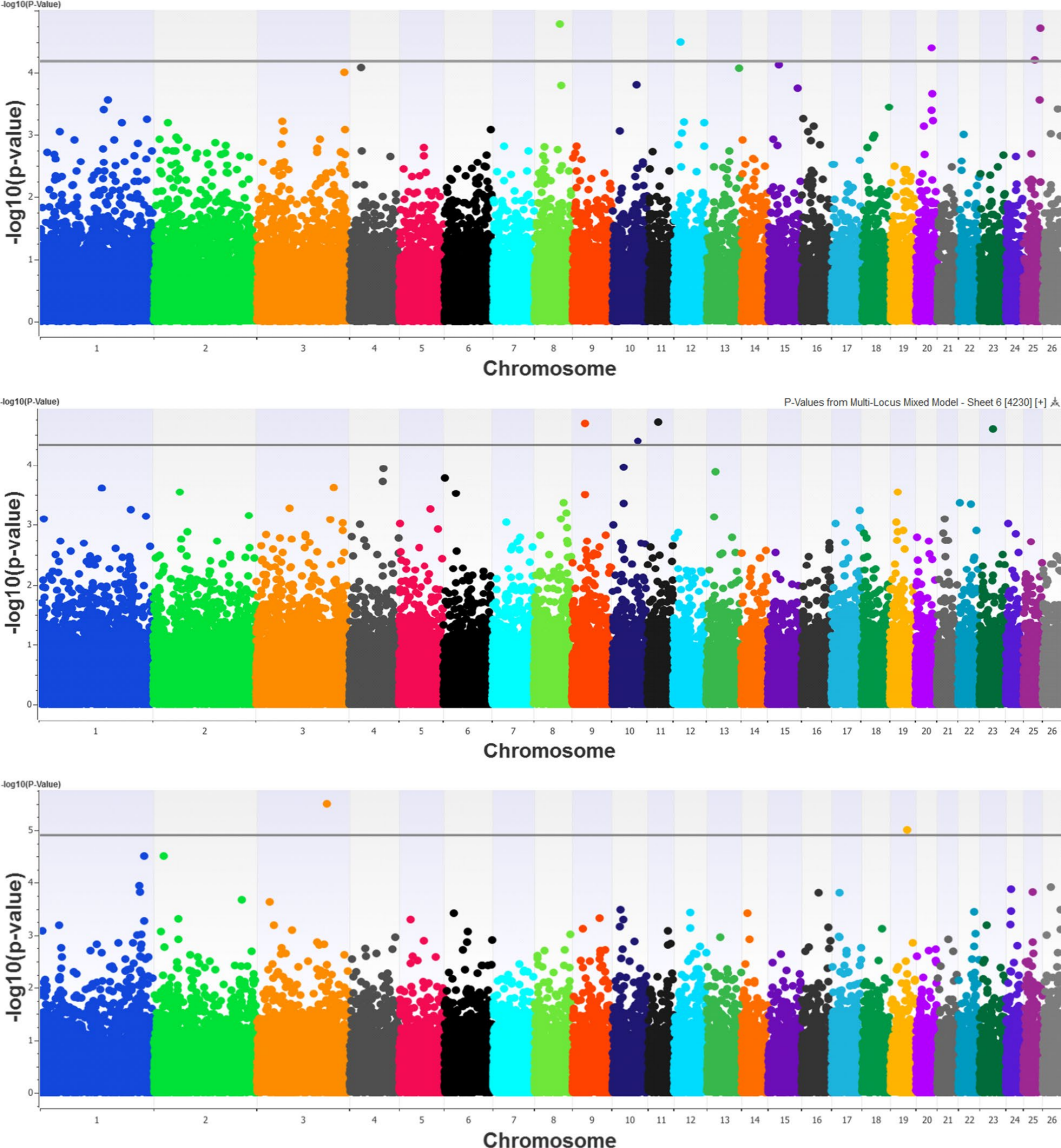
Manhattan plots representing chromosome-wide associations with the three body size principal components (PC1 *top*, PC2 *middle* and PC3 *bottom plot*) in Frizarta sheep. SNP −log10(p values) are shown across the 26 autosomal chromosomes. *Horizontal lines* denote significance threshold

Figure 2 shows the profiles of the p values (presented as −log10) for all SNPs across the 26 autosomes. No SNPs were significant at the genome-wide level (p < 0.05) after applying the Bonferroni or the FDR correction. However, 11 chromosome-wide significant (p < 0.10, both criteria) SNPs were identified, five for PC1, four for PC2 and two for PC3. A detailed description of the significant SNPs is provided in Table 3. Taken together, the SNPs explained jointly 0.179 of the phenotypic variance of the ‘width Dimension’ factor (PC1), 0.142 of the ‘height Dimension’ factor (PC2) and 0.089 of the ‘length Dimension’ factor (PC3). Furthermore, the fraction of the phenotypic variance that is explained by the empirically estimated genomic relatedness matrix, called pseudo-heritability [33], was as high as 46, 76 and 74% for the three PC, respectively, with 38, 36 and 44% of these variances attributed to the SNPs, respectively (results not shown).

**Table 3:** SNPs that are significantly associated with the three body principal components in Frizarta dairy ewes

SNP	Chr^a^	Position	p value	−log10 (p value)	−log10 (p value) expected	p_BON_^b^	p_FDR_^c^	pve^d^
PC1, width dimension								
OAR8_65677467.1	8	60857281	1.67E−05	4.778	4.936	0.028	0.028	0.040
s16706.1	25	40519001	1.91E−05	4.718	4.458	0.016	0.016	0.039
s75176.1	12	15687883	3.18E−05	4.498	4.237	0.045	0.045	0.037
s09443.1	25	26277908	6.34E−05	4.198	3.981	0.053	0.027	0.035
OAR20_41133825.1	20	37570240	3.97E−05	4.401	4.091	0.038	0.038	0.028
PC2, height dimension								
s49406.1	11	27245550	1.96E−05	4.707	4.936	0.019	0.019	0.039
OAR23_33457070.1	23	31731118	2.58E−05	4.589	4.185	0.025	0.025	0.038
OAR10_65976077.1	10	63659341	4.03E−05	4.394	4.091	0.060	0.060	0.036
OAR9_32819540.1	9	31286828	2.10E−05	4.677	4.458	0.037	0.037	0.028
PC3, length dimension								
s19829.1	3	168409560	0.32E−05	5.497	4.936	0.013	0.013	0.047
OAR19_41234161.1	19	39335737	0.99E−05	5.002	4.458	0.010	0.010	0.042

^a^Ovine chromosome

^b^Bonferroni p value

^c^False discovery rate p value

^d^Proportion of variance explained

### Previously reported QTL

Table 4 summarizes the previously published sheep QTL that lie within 1-Mb regions around the significant SNPs and are reported as either meat, production or reproduction QTL. Note that all the QTL are related to body size (foreleg length, jaw length) or body weight traits (carcass weight, fat weight, etc.) and this is also valid for the three reproduction QTL that refer to testes weight. From the 11 significant SNPs, the 1-Mb regions around only one of these i.e. OAR19_41234161.1 (OAR19, PC3) did not harbor any reported QTL. The largest number of QTL was found for SNP OAR23_33457070.1 (OAR23, n = 8), followed by SNP s49406.1 (OAR11, n = 6) and SNP OAR10_65976077.1 (OAR10, n = 5) and are associated with height dimension (PC2).

**Table 4:** Previously published body size or body weight related QTL located within 1 Mb from SNPs that are significantly associated with body size in Frizarta sheep

SNP	Chr^a^	QTL type	QTL	QTL ID [28]
PC1, width dimension				
OAR8_65677467.1	8	Meat_QTL	Internal fat amount	14288
s75176.1	12	–	–	–
OAR20_41133825.1	20	Meat_QTL	Ultrasound fat depth	13705
		Meat_QTL	Foreleg length	13795
s16706.1	25	Reproduction_QTL	Testes weight	12925
s09443.1	25	Reproduction_QTL	Testes weight	12925
PC2, height dimension				
OAR9_32819540.1	9	Meat_QTL	Hot carcass weight	14290
		Meat_QTL	Longissimus muscle area	14323
OAR10_65976077.1	10	Reproduction_QTL	Testes weight	12923
		Meat_QTL	Lean meat yield percentage	14295
		Meat_QTL	Carcass fat percentage	14294
		Meat_QTL	Carcass bone percentage	14293
		Meat_QTL	Fat weight in carcass	14292
s49406.1	11	Production_QTL	Body weight (slaughter)	14297
		Meat_QTL	Internal fat amount	14298
		Meat_QTL	Jaw length	13802
		Meat_QTL	Jaw length	14178
		Meat_QTL	Hot carcass weight	14296
		Production_QTL	Average daily gain (birth-43 weeks)	13966
OAR23_33457070.1	23	Production_QTL	Body weight	16039
		Production_QTL	Total fat	14331
		Production_QTL	Body weight (slaughter)	14312
		Production_QTL	Total fat	14335
		Meat_QTL	Lean meat yield percentage	14274
		Meat_QTL	Hot carcass weight	14311
		Meat_QTL	Lean meat yield percentage	14314
		Meat_QTL	Carcass fat percentage	14313
PC3, length dimension				
s19829.1	3	Meat_QTL	Internal fat amount	14014
OAR19_41234161.1	19	–	–	–

^a^Ovine chromosome where the marker is located

### Positional candidate genes and gene prioritization analysis

A total of 197 positional candidate genes located in the 1-Mb regions around significant SNPs were identified on the annotated ovine genome (see Additional file 1). The largest number of these genes (n = 97) were located on OAR11, followed by genes on OAR25 (n = 45), OAR3 (n = 14), OAR8 (n = 12), OAR20 (n = 8), OAR23 and OAR9 (n = 7), OAR19 (n = 6) and OAR10 (n = 1). No genes were found in the 1-Mb regions around the SNP on OAR12. Seven SNPs were included within ovine annotated genes i.e. *PTPRG*, *ZNF521*, *PDE7B*, *LRRC20*, *GRID1*, *ANKS1B* and *ALOX12B*. Table 5 shows the results of gene prioritization analysis according to the semantic annotation imposed. From the initial 197 positional candidate genes, 160 could be used in the prioritization analysis whereas the remaining 37 genes were not sufficiently annotated to identify the homologous human genes. From the 160 positional candidate genes submitted to the prioritization analysis, 39 had significant functional association/relevance to the traits of interest (p < 0.05; Table 5). This list of functional candidate genes includes at least one gene for each SNP except for s75176.1 (OAR12) and OAR10_65976077.1 (OAR10).

**Table 5:** List of prioritized ovine genes based on guilt by association prioritization analysis

Gene	Overall rank^a^	Rank in SNP	p value^b^	Chr^c^	Marker	Minimum^d^ distance (kb)	PC^e^
*TP53*	1	1	0.003	11	s49406.1	338	2
*BMPR1A*	2	1	0.005	25	s16706.1	353	1
*PIK3R5*	3	2	0.007	11	s49406.1	635	2
*RPL26*	4	3	0.008	11	s49406.1	254	2
*PRKDC*	5	1	0.009	9	OAR9_32819540.1	876	2
*NODAL*	6	1	0.01	25	s09443.1	69	1
*PRF1*	7	2	0.01	25	s09443.1	220	1
*COL13A1*	8	3	0.014	25	s09443.1	318	1
*HK1*	9	4	0.014	25	s09443.1	816	1
*APAF1*	10	1	0.014	3	s19829.1	696	3
*MYH10*	11	4	0.014	11	s49406.1	339	2
*POLR2A*	12	5	0.017	11	s49406.1	482	2
*DVL2*	13	6	0.017	11	s49406.1	707	2
*CHRNB1*	14	7	0.02	11	s49406.1	523	2
*CTC1*	15	8	0.021	11	s49406.1	123	2
*AHI1*	16	1	0.021	8	OAR8_65677467.1	371	1
*SLC2A4*	17	9	0.023	11	s49406.1	665	2
*LDB3*	18	2	0.023	25	s16706.1	273	1
*PEX7*	19	2	0.024	8	OAR8_65677467.1	914	1
*GUCY2D*	20	10	0.025	11	s49406.1	48	2
*MAP3K5*	21	3	0.026	8	OAR8_65677467.1	654	1
*NHLRC1*	22	1	0.028	20	OAR20_41133825.1	709	1
*EIF4A1*	23	11	0.028	11	s49406.1	414	2
*ALOXE3*	24	12	0.028	11	s49406.1	15	2
*FHIT*	25	1	0.029	19	OAR19_41234161.1	785	3
*NTN1*	26	13	0.031	11	s49406.1	744	2
*HES7*	27	14	0.035	11	s49406.1	39	2
*TMEM107*	28	15	0.036	11	s49406.1	95	2
*PSMA8*	29	1	0.036	23	OAR23_33457070.1	813	2
*ATP1B2*	30	16	0.036	11	s49406.1	355	2
*ACADVL*	31	17	0.036	11	s49406.1	715	2
*FGF11*	32	18	0.037	11	s49406.1	533	2
*MYB*	33	4	0.037	8	OAR8_65677467.1	655	1
*TNFSF12*	34	19	0.038	11	s49406.1	434	2
*TNK1*	35	20	0.04	11	s49406.1	582	2
*ADAMTS14*	36	5	0.041	25	s09443.1	265	1
*ALOX12B*	37	21	0.042	11	s49406.1	0	2
*MMRN2*	38	3	0.044	25	s16706.1	500	1
*ZNF521*	39	2	0.049	23	OAR23_33457070.1	0	2

^a^Gene rank after prioritization analysis

^b^p value from prioritization analysis

^c^Ovine chromosome

^d^minimum distance from marker

^e^Principal component

There were 14 positional candidate genes for SNP s19829.1 on OAR3 and only the *APAF1* gene was suggested as a functional candidate based on PA and ranked 12th overall, while three of the other genes (*ANKS1B*, *SCYL2* and *NR1H4*) were associated with relevant mammalian phenotypes (see Additional file 2). Among these, the *ANKS1B* gene also included the SNP. Twelve positional candidate genes were found in the 1-Mb regions around SNP OAR8_65677467.1 on OAR8 with four of them (*AHI1*, *PEX7*, *MAP3K5* and *MYB*) suggested as functional candidates by PA (Table 5) and five (*BCLAF1*, *AHI1*, *MYB*, *HBS1L* and *PEX7*) being associated with relevant mammalian phenotypes (see Additional file 2). Notably, the chromosomal region around SNP OAR8_65677467.1 contained the highest percentage of prioritized positional candidate genes. One gene i.e. *BCLAF1* located in this region and associated with relevant phenotypes was not included in the prioritized gene list.

For SNP OAR9_32819540.1 on OAR9, seven positional candidate genes were identified with *PRKDC* being the only prioritized gene (Table 5). This gene is associated with relevant mammalian phenotypes (see Additional file 2). The only positional candidate gene (*SLITRK5*) for SNP OAR10_65976077.1 on OAR10 was neither included in the prioritized list after PA nor associated with relevant mammalian phenotypes. For SNP s49406.1 on OAR11, 97 positional candidate genes were detected of which 21 were also prioritized (Table 5). Among these 21 genes, 12 (*ALOXE3*, *HES7*, *PER1*, *CTC1*, *PFAS*, *TP53*, *MYH10*, *POLR2A*, *TMEM256*, *SLC2A4*, *CLDN7* and *DLG4*) are known to be associated with relevant mammalian phenotypes (see Additional file 2) and nine (*BCLAF1*, *GRID1*, *SGPL1*, *NEUROG3*, *PER1*, *SOX15*, *CD68*, *ANKS1B*, and *FEZF2*) were not in the prioritization gene list, although they are associated with relevant mammalian phenotypes in the Mouse Genome Informatics (MGI) database [34]. The top ranking gene in the prioritization list was *TP53*, which is associated with related mammalian phenotypes and has been reported as a candidate gene for body conformation traits in a GWAS (see “Discussion” section). Interestingly, the *PIK3R5* and *RPL26* genes that ranked second and third, respectively, based on the PA showed no obvious functional link with BS.

Among the six positional candidate genes identified in the 1-Mb regions around SNP OAR19_41234161.1 on OAR19, only *FHIT* was also suggested as a functional candidate gene (Table 5). To date, *FHIT* has not been reported to be associated with relevant mammalian phenotypes (MGI database search). On the contrary, *FEZF2*, which was not included in the prioritization list, is associated with related phenotypes (see Additional file 2). For SNP OAR20_41133825.1 on OAR20, eight positional candidate genes were detected, among which *NHLRC1* was included in the prioritized list (Table 5). The *NHLRC1* gene is associated with relevant phenotypes (see Additional file 2) and two of the remaining non prioritized genes (*ID4* and *TPMT*) were also associated with relevant phenotypes (see Additional file 2). For SNP OAR23_33457070.1 on OAR23, seven positional candidate genes were detected, two of which were in the prioritization list (*PSMA8* and *ZNF521*; Table 5) but only *ZNF521* is associated with related phenotypes (see Additional file 2). Two significant SNPs were identified on chromosome OAR25, s16706.1 and s09443.1. For SNP s16706.1, 21 positional candidate genes were detected, among which *LDB3* and *BMPR1A* were both in the prioritization list (Table 5) and associated with related phenotypes (see Additional file 2). *GRID1*, although not among the prioritized genes, is associated with relevant phenotypes. For SNP s09443.1, 24 positional candidate genes were found, among which five were in the prioritization list. Four of these prioritized genes and three from the remaining positional candidates were associated with relevant MGI-retrieved phenotypes (see Additional file 2). *NODAL* was both the top ranking gene among the prioritized genes (Table 5) and the most closely positioned to the respective SNP (69 kb). Finally, no positional candidate genes were identified for SNP s75176.1 on OAR12.

A few of the prioritized candidate genes either harbor (*ZNF521* and *ALOX12B*) or are located in close vicinity to the respective significant SNP (e.g. 15 kb-*ALOXE3*, 40 kb-*HES7*, 48 kb-*GUCY2D*) whereas the top five ranked genes (*TP53*, *BMPR1A*, *PIK3R5*, *RPL26* and *PRKDC*) are more distantly located with distances from the significant SNP ranging from 254 to 876 kb. Genes that ranked first for each significant SNP (*APAF1*, *AHI1*, *PRKDC*, *TP53*, *FHIT*, *NHLRC1*, *PSMA8*, *NODAL* and *BMPR1A*) were all located at distances greater than 50 kb from the respective SNPs with distances ranging from 69 to 876 kb.

## Discussion

In this study, we show that the use of PCA is an efficient variable reduction method that resulted in three interpretable PC, which captured a significant part of the phenotypic variance of the original 10 variables. This made it possible to carry out three GWAS instead of 10 while, at the same time, increasing the power of the study. Another useful implication of using the PC instead of the original variables relates to the interpretation of the results. Apart from describing body size (height, width and length), the three PC can be used to describe body shape and body volume as well. Specifically, the pairwise PC combinations i.e. PC1–PC3 and PC2–PC3 define the body shape from above and laterally, respectively, while the three PC jointly describe the body volume of an animal. This means that results on individual PC can be combined to make inferences on genes that affect body size or body volume, as well. Finally, since body measurements explain a significant amount (68.5%) of the variation in body weight (BW) in this breed [35], our results are also useful to suggest candidate genes for BW as well.

The search for putative genes within defined regions (±1 Mb) around the significant SNPs provided a considerably large number (n = 197) of positional candidate genes. This rendered the discovery of plausible causative genes a real challenge. In almost all GWAS, the genes that lie in closest proximity (e.g. 100 kb) to the significant SNPs combined with information on the functional relevance to the traits studied are considered as the most plausible causative candidate genes. When no or only limited information on functional relevance exists, inference on functional candidates is based solely on their proximity to the markers. However, proximity does not guarantee functional relevance and it is most probable that causative candidate genes also exist among distantly located loci. In our case, using only the functional relevance criterion [MGI retrieved phenotypes, (see Additional file 2)] resulted in a significantly smaller number (41 of 197) of functional candidate genes, nine of which were in closest proximity (100 kb) to the respective SNPs, i.e. *GRID1*, *ZNF521*, *ANKS1B*, *ALOXE3*, *HES7*, *PER1*, *NODAL*, *VAMP2* and *NPFFR1*. When only the distance criterion was imposed (100 kb), the number of positional candidate genes further decreased to 18 (*PDE7B*, *LRRC20*, *GRID1*, *ZNF521*, *ALOX12B*, *PTPRG*, *ANKS1B*, *ALOXE3*, *ALOX15B*, *HES7*, *EIF4EBP2*, *GUCY2D*, *LOC106991397*, *PER1*, *NODAL*, *VAMP2*, *NPFFR1* and *TMEM107*) with, as previously described, only half of them being associated with related phenotypes.

**Fig. 3:**
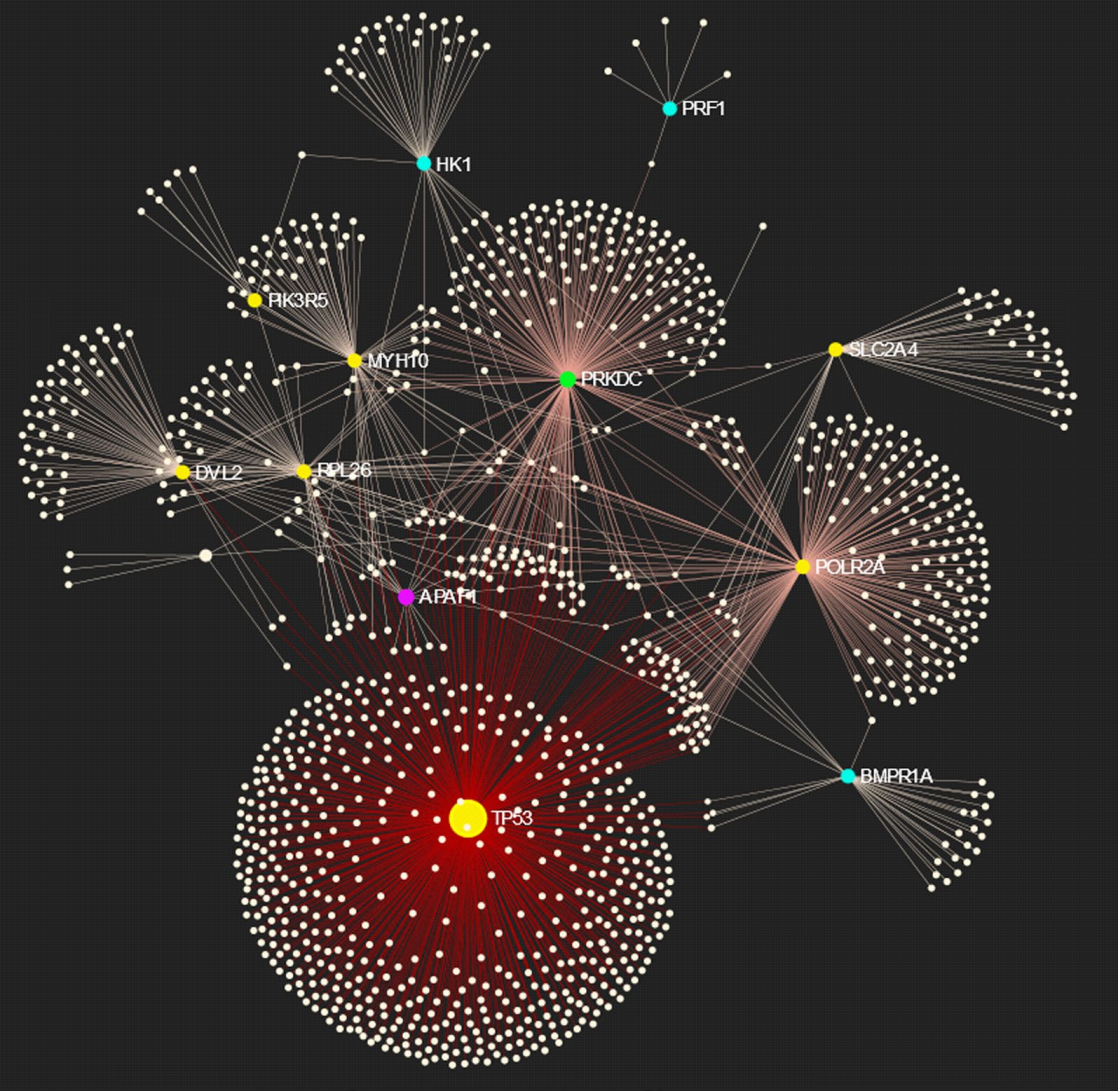
Depiction of a network with connections of the top 20 prioritized genes. The network is comprised of 1190 nodes, 1430 edges and 16 seed proteins. Genes are shown in yellow (OAR11), blue (OAR25), green (OAR9) or magenta (OAR3). *White colors* represent connected genes and edges number of associations. Network analysis was performed via the web application NetworkAnalyst [64–66] and the network interactome database innateDB [67] comprising literature curated comprehensive protein–protein interaction (PPI) data (~140,000 interactions) [68]. Here, genes were prioritized using the degree of centrality

Given the limitations of using either the annotated function criterion including phenotypes of the genes or the criterion of relative position to the significant SNPs, an alternative strategy to identify causative candidate genes was used here based on the GBA principle. This principle states that genes, which are associated or interacting with each other, are more likely to share a phenotype, function or pathway. Gene PA is then based on functional relevance by incorporating all the available annotation data as well as known protein–protein network interactions collected from the numerous reports on associations and from high-throughput data. Taken together, these data often build large interaction networks in which assignment of gene function is achieved by using machine-learning approaches [36]. Figure 3 graphically shows how this procedure works for the top 20 prioritized genes.

Based on the PA described above, the original 197 candidate genes were reduced to 39 plausible candidate genes. This significant reduction in number and increase in the functional relevance of the candidate genes should result in significantly reduced costs, time and labor required for further downstream validation of the prioritized candidates. The prioritized genes spanned the whole range (0 to 1 Mb) of the genomic regions searched while only about half of them (n = 20) had related phenotypes in the MGI retrieved phenotypes. The validity of the prioritization method proposed here was questioned in the case of genes that were prioritized but were not associated with known phenotypes (*MAP3K5*, *NHLRC1*, *MMRN2*, *ADAMTS14*, *ALOX12B*, *GUCY2D*, *TMEM107*, *RPL26*, *ATP1B2*, *EIF4A1*, *TNFSF12*, *CHRNB1*, *FGF11*, *TNK1*, *PIK3R5*, *NTN1*, *PSMA8*, *APAF1* and *FHIT*). This was an intriguing question since 15 of the above 19 genes were located in genomic regions that are reported to harbor meat or production QTL (see Additional file 1). Our results show that PA was indeed helpful in identifying functional candidate genes that would be otherwise, overseen, due to absence of functional relevance based on reported mammalian phenotypes. The *PIK3R5*, *RPL26*, *PSMA8* and *APAF1* genes fall within this category since they are not associated with relevant mammalian phenotypes but they were highly prioritized either overall or within the respective SNP. PIK3R5, is a regulatory subunit of the class I phosphatidylinositol 3-kinase (PI3K) gamma complex and it has been shown that mutations in another PI3K regulatory gene subunit, *PIK3R1*, are responsible for human short syndrome [37–39], which is characterized by a variety of symptoms including short stature. Moreover, the PI3K signaling pathway has been implicated in growth hormone and insulin resistance [40]. Ribosomal protein L26 (RPL26) is a positive regulator of *TP53* [41], a gene that as described below has been identified as a candidate gene for body conformation traits. In addition, human mutations in *RPL26* are associated with diamond-blackfan anemia, a syndrome that includes growth retardation and skeletal abnormalities [42]. For the first time in a GWAS, *PSMA8*, the top ranking functional candidate gene located 813 kb away from SNP OAR23_33457070.1 is associated with BS traits. Although no direct evidence links *PSMA8* with BS traits, a gene encoding a similar protein, *PSMA1*, was identified by Saatchi et al. [43] as a candidate gene for body conformation traits in beef cattle. Finally, the prioritization of the *APAF1* gene is justified since it has been shown that APAF1-deficient mice were smaller and had lower levels of growth hormone compared to wild type littermates [44].

Furthermore, GBA-based PA has proved to be useful to significantly reduce the number of functional candidate genes when multiple candidate genes were present within the 1-Mb regions around a SNP. A good example here was *HES7*, the closest gene to SNP s49406.1 (OAR11). Mutations in *HES7* cause spondylocostal dysostosis in humans [45] and dogs [46], short-tailed trait in cats [47, 48] and affect skeleton formation [49] as well as body length in mice [50]. Nevertheless, based on PA, *HES7* ranked 14^th^/21 for this SNP, while more distantly located genes from the same SNP ranked first (*TP53*, 338 kb), third (*PIK3R5*, 635 kb) and fourth (*RPL26*, 254). A careful review of the available literature strengthens the prioritization of *TP53* over *HES7*. In addition to relevant mouse mutant phenotypes (see Additional file 2) and [51–54], *TP53* polymorphisms are associated with human birth weight [55], with mature size in sheep [13] and with human height [56].

The validity of the GBA-based PA is further strengthened by the presence in the prioritization list of genes that, based on available data, are very strong functional candidates for BS traits. For example, *BMPR1A* that ranked second overall is essential for embryogenesis [57], including skeletogenesis, and postnatal bone homeostasis [58]. Expression of *BMPR1A* was shown to be downregulated in a mouse model for human idiopathic proportionate short stature [59]. It is also involved in the regulation of adipogenesis and variants of *BMPR1A* are associated with human obesity [60]. Finally, another gene encoding a BMP2/4 receptor, *BMPR1B* was identified as a candidate gene for variation in mature size in sheep [13]. Note that the five top prioritized genes (*TP53*, *BMPR1A*, *PIK3R5*, *RPL26* and *PRKDC*) are depicted as nodes with a large number of connections (Fig. 3).

Although GBA-based PA has proved to be useful in cases such as those described above, it is not a panacea and it cannot be universally applied, especially in the case when genes with unknown functions are part of a gene network. In GBA-based networks, there is a highly statistically significant relationship between shared Gene Ontology annotations and network edges [36], which means that high node-degree genes tend to have many functions as well. As may be reasonably expected, such genes are expected to show a good performance during gene function prediction without using information on which genes they are associated with [36]. Such a scenario may explain why GBA-based PA ranked as highest the three genes *PIK3R5*, *RPL26* and *PRKDC* although they have no obvious relation to the phenotypes studied here. At the other extreme, a poor prediction performance for gene function should be anticipated for genes with limited annotated functions. This may be the reason why none of the genes *BCLAF1*, *HBS1L*, *ID4*, *TPMT*, *GRID1*, *NPFFR1*, *SGPL1*, *NEUROG3*, *PER1*, *VAMP2*, *PFAS*, *SOX15*, *CD68*, *TMEM256*, *PLSCR3*, *CLDN7*, *DLG4*, *ANKS1B*, *SCYL2*, *NR1H4* and *FEZF2*, which were found to be related to MGI phenotypes and located on relevant QTL, were not highly prioritized here. Apart from the amount of information (annotated functions), one should also bear in mind that PA (as well as network analysis) such as that used here, are based on protein–protein interaction (PPI) databases that specifically refer to human proteins. Information on other species such as the mouse or other mammalian species, including the livestock species, may not be incorporated in these databases. In addition, as Gillis and Pavlidis [36] emphasized, a more detailed and systematic encoding of gene function in networks should be pursued, since functional information within gene networks depends on specific and critical interactions.

Finally, the positional candidate genes listed in the current study were compared with candidate genes identified by other GWAS for body composition traits in sheep [7, 12, 13], cattle [43, 61, 62] and humans [63], which led to the identification of eight common gene candidates: *GRID1*, *ALOX12*, *SLC16A13*, *SLC16A11* [13], *TP53* [13, 56], *STX8* [7, 61], *NTN1* [7], and *ZNF521* [62], among which three, i.e. *TP53*, *NTN1* and *ZNF521*, were also identified as functional candidates based on the PA.

## Conclusions

In conclusion, our results provide both novel causative candidate genes and support for previously identified candidate genes from other GWAS for BS traits in sheep. Using a larger sample of animals would improve the power of the study and the identification of candidate causative genes. Gene prioritization methods have proved to be useful in identifying SNPs/genes with increased biological relevance and in enriching signals in GWAS but they are subject to certain limitations. New gene prioritization methods are needed that would generate biologically plausible candidate genes by incorporating all available biological information.

## Additional files

**Additional file 1.** Positional candidate genes located within 1 Mb from significant markers.

**Additional file 2.** Body size related mammalian phenotypes associated with positional candidate genes.
